# Predicting responses to warming temperatures for early growth rate, size and age at sexual maturity and mortality in male *Xiphophorus multilineatus*

**DOI:** 10.7717/peerj.21555

**Published:** 2026-07-22

**Authors:** Keith Tompkins, Donald B. Miles, Molly R. Morris

**Affiliations:** Department of Biological Sciences, Ohio University, Athens, OH, United States of America

**Keywords:** Temperature-size rule, Growth-mortality tradeoff, *Xiphophorus multilineatus*, Alternative reproductive tactics

## Abstract

The majority of ectotherms conform to the temperature-size rule, a reaction norm where individuals reared in colder environments grow slower, take longer to reach sexual maturity and attain a larger adult size than conspecifics from warmer environments. In relation to the temperature-size rule which influences growth rates, tradeoffs often exist between growth rate and survival across taxa, namely increased growth rate frequently leads to a higher probability of reaching sexual maturity but a shorter adult life span. We examined whether the temperature-size rule applies to one of the two male alternative reproductive tactics (ARTs) in *Xiphophorus multilineatus*. Sibling males from the courter ART were reared at one of two temperatures (25 °C and 20 °C) and monitored through sexual maturity. Males followed the temperature-size rule of faster growth and earlier maturation at a smaller size in the warm environment. While early growth rates and mortality were higher in the warm treatment, we did not detect an influence of growth rate on mortality when controlling for temperature and dam. We did detect, however, evidence of dam-associated variation in size at sexual maturity, but not age, with potential implications for the mechanisms impacting the onset to puberty. Finally, depending on whether the smaller sneaker males also conform to the temperature-size rule, we discuss how tradeoffs that help to maintain the two male ARTs in *X. multilineatus* (age and size at sexual maturity, growth-mortality rate tradeoff) might be altered by warmer temperatures.

## Introduction

Temperature is one of the most important factors influencing development and growth rates, and therefore understanding its influence on developmental traits has become integral to understanding the effects of increasing global temperatures on population dynamics (Beltrán et al., 2021; Crabbe, 2008; Iltis et al., 2019; Ohlberger, 2013). Growth rate and adult size are influenced by environmental temperature in a similar way across the majority of ectotherms: species with broad geographic ranges develop thermal clines in adult body size where individuals from colder environments grow slower and attain larger adult size than those from warmer environments (Angilletta, Steury & Sears, 2004). This is a form of phenotypic plasticity referred to as the “temperature-size rule” (Atkinson, 1994). While a review of 92 species of ectotherms, including plants, protists and animals, found that approximately 80% demonstrated the pattern of slower growth and larger adult size among species occurring in colder environments and faster growth, smaller adult size for species in warmer environments (Atkinson, 1994), exceptions include diatoms, that show no consistent relationship between size and temperature (Adams et al., 2013), many species of lizards that exhibit the reverse of the temperature-size rule attributed to their ability to behaviorally thermoregulate (Sears & Angilletta Jr, 2004), and insects that do not always conform to the temperature-size rule due to the influence of generation time and length of growing season on body size (Fischer & Fielder, 2002).

There are both costs and benefits of responding to warmer temperatures by following the temperature-size rule that are important to examine to not only predict the selection on species responses to increasing global temperatures, but also to better understand the mechanisms behind these responses. For example, for aquatic organisms, the relatively low availability of oxygen in in warmer waters would favor a smaller body size with a concomitant lower metabolic oxygen demand (Forster, Hirst & Atkinson, 2012; Verberk et al., 2021). In other words, the temperature-size rule may be a phenotypically plastic response that provides a safeguard against getting too large in an environment where oxygen is more likely to be limiting. However, controversy over the validity of this mechanism remains (Audzijonyte et al., 2019). Faster growth rates leading to attainment of sexual maturity at smaller sizes can also be beneficial by increasing the probability of reaching sexual maturity given a high risk of predation (Dmitriew, 2011; Takeshita, 2024). However, the faster growth rates associated with reaching sexual maturity at smaller sizes may present a cost in relation to a tradeoff between growth rate and survivorship (Mangel & Stamps, 2001; Lee, Monagha & Metcalfe, 2013; Takeshita, 2024). This relationship can be attributed to the need for increased foraging for resources to support the cost of faster growth, leading to a risk of mortality associated with the increased time of exposure to potential predators during prolonged bouts of foraging. The growth rate-mortality tradeoff has also been linked to cost that are not associated with increased predation (Metcalfe & Monaghan, 2003), including increased developmental errors at the cellular level (Arendt, 1997; Jimenez, 2018), diverting energy from immune responses and other physiological stress responses (Mangel & Stamps, 2001; Van der Most et al., 2011), and increased production of reactive oxygen species (ROS), resulting in oxidative damage to cells and thus shorter lifespans (Mangel & Munch, 2005). Finally, maturing at smaller sizes is often associated with lower fitness in systems where male-male competition and female mate preference select for larger male size.

**Figure 1:**
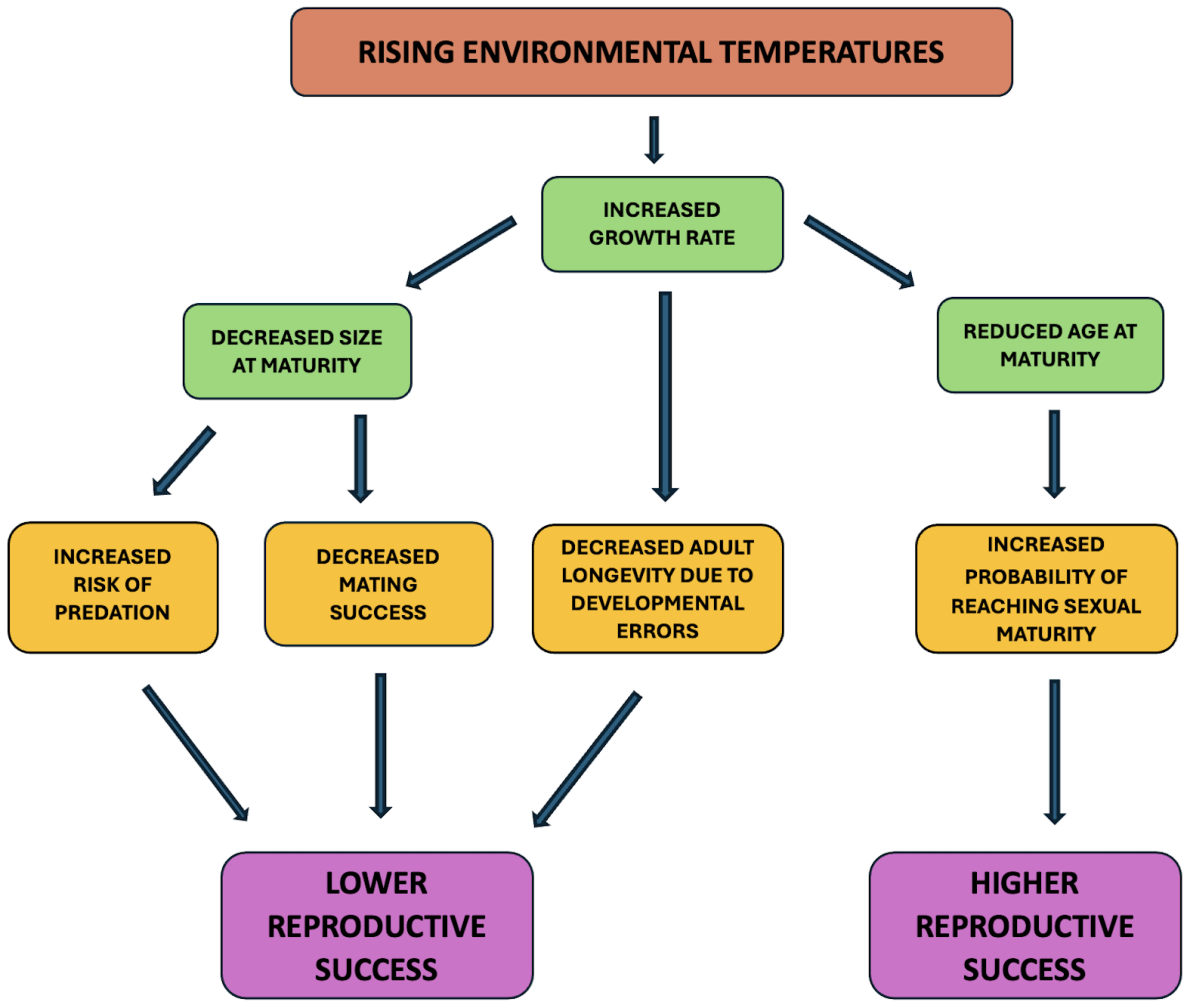
The effects of environmental warming on growth, size and age at sexual maturity reflecting the temperature-size rule. Subsequent effects on survival and reproduction, both potential and known for this system, are indicated in the orange and purple squares. Decreased size at sexual maturity = smaller adult size as swordtail males do not continue to grow after sexual maturity (Lampert et al., 2010). Smaller *Xiphophorus* males have a higher risk of predation (Basolo & Wagner Jr, 2004), and decreased mating success in several species of swordtails including *X. multilineatus* (*e.g.* Prenter, Taylor & Elwood, 2008; Rios-Cardenas & Morris, 2011). Increased growth rates have been shown to be correlated with decreased adult longevity in *X. multilineatus* (Morris et al., 2016; Weinstein et al., 2019). Given any level of juvenile mortality, reaching sexual maturity sooner increases probability of reaching sexual maturity, a pattern also detected in the wild for *X. multilineatus* (Weinstein et al., 2019).

We examined whether the males from the courter ART in *X. multilineatus* would respond to warming temperatures by following the temperature-size rule. Given that this is a species for which several of the costs and benefits of growth rates and body size have been previously determined (Fig. 1), following the temperature-size rule will allow for predictions as to how this species would respond to global warming. The two ARTs in *X. multilineatus* (courter and sneaker) are dimorphic for size at sexual maturity when males stop growing, a difference that is genetically influenced by variation in copy number of *mc4r* B alleles on the Y chromosome (Lampert et al., 2010). Differences in size at sexual maturity are detected not only between the ARTs, but within the courter ART due to extensive variation in copy number of *mc4r* on the Y chromosome, which appears to be involved in puberty signaling (Liu et al., 2019; Liu et al., 2020). The maintenance of the genetic polymorphism (ARTs) as well as genetic variation within the courter males has been examined in relation to the tradeoff between having a higher probability of reaching sexual maturity (maturing earlier as a small male, sneakers), compared to the benefits of maturing later as a larger male (courters) and gaining more mates (Rios-Cardenas, Bono & Morris, 2018). However, previous studies have also detected the intrinsic trade-off between fast growth rate and increased adult mortality for *X. multilineatus* courter males in both a laboratory setting (Morris et al., 2016) and the field (Weinstein et al., 2019). Therefore, an additional tradeoff influencing variation in size in this species that this study can address is the growth-mortality tradeoff (Mangel & Stamps, 2001), where even though individuals that grow faster are more likely to survive to adulthood, they are less likely to persist in the adult population of males (Weinstein et al., 2019; Abbott, Rios-Cardenas & Morris, 2019).

In the current study, we used a split-brood design to address the following questions. (1) How does rearing temperature influence early growth rate, size at sexual maturity and age at sexual maturity in *X. multilineatus* courter males, while controlling for maternal effects? Given that males follow the temperature-size rule, warming temperatures will result in faster growing smaller courter males in this species, with the given costs and benefits associated with faster growth and smaller adult size. (2) Does dam (maternal effects) influence variation in early growth rates, size or age at sexual maturity? (3) Does rearing temperature, early growth rate and/or dam influence mortality in courter males? Sufficient variation across dams in the responses to warmer temperatures could be due to maternal provisioning leading to plasticity in responses as well genetic differences suggesting the potential for warming temperatures to select for the evolution of more optimal responses as well. Finally, depending on whether the males from the sneaker ART also respond to warmer temperatures by following the temperature-size rule as well, we discuss how warming temperatures could potentially influence the evolutionary balance of the two ARTs in this species.

## Material and Methods

Males used to test the effects of rearing temperature on growth were obtained by isolating females from an existing community tank that included only courter male *X. multilineatus* and was located in the Morris Lab at Ohio University. Eighteen out of 25 females isolated dropped fry. Fry remained in their natal tank until age 30 d to avoid potentially harming the fish by moving them too soon (Marcus & McCune, 1999). Fry were photographed at 30 days of age and then broods were split between temperature treatments, randomly choosing which individuals to transfer to the 2.5 l tanks in respective environmental chambers: 72 fry in warm (25 °C), 72 fry in cold (20 °C). Location of tanks were similar across racks and shelves within each of the environmental chambers. Temperatures were chosen to be 2.5 °C above and 2.5 °C below the average laboratory temperature (22–23 °C) where parents were reared, reducing the possibility of excessive mortality at more extreme temperatures. We did not include a third group reared at an intermediate temperature for two reasons. First, factors that could potentially influence growth rates were controlled for across the two temperature treatments. Second, including a third group reared at the same temperature as the main laboratory would have reduced the number of fry assigned to each treatment. Comparisons to a previous study (Murphy, Goedert & Morris, 2014) where courter males were reared at 22 °C are made where appropriate (size and age at sexual maturity, but not growth rates per se). All fry were fed a diet of Ken’s^®^ spirulina flake food in the morning daily, and brine shrimp in the afternoon five days a week. We minimized the welfare impacts on subjects by providing environmental enrichment of artificial plants and gravel substrate in each individual tank. Fish were kept on a 12:12-hour light: dark cycle. Each tank had its own filter, which removed any water transfer from tank to tank. Dissolved oxygen saturation in tank water between cold and warm treatments were similar (*N* = 8, Cold = 100.8 ± 1.1% dissolved oxygen; *N* = 8, Warm = 97.7 ± 1.4% dissolved oxygen). The optimal dissolved oxygen saturation for tropical freshwater fish is between 80–100% (Mallya, 2007). Standard length (SL) was used for all size measurements, which is the measure from the tip of the snout to posterior edge of the caudal peduncle (Basolo, 1990). ImageJ software was used to measure SL (mm), and the student that assisted in measuring size from the photos was unaware of treatment groups. All statistics were calculated using R software version 4.3.2 (R Core Team, 2023). Individuals excluded from analyses were those that died prior to the measurement being analyzed or did not have a sibling in both temperature treatments for the mortality analysis. Housing of the fish in individual tanks continued until age 765 days, when remaining fish were moved to group breeding tanks in the main laboratory.

### Early growth

Early growth rate was calculated as the difference in length between 30 and 100 days old (when they could be safely moved to the temperature treatments and prior to sexual maturity for all individuals) divided by the duration (70 days) and reported as mm/d. To determine factors influencing variation in early growth rate, we used a linear mixed-effects model (function “lmer” in the package lme4; Bates et al., 2015) with treatment as a fixed effect, initial size (SL) as a covariate and dam as a random effect. Initial body size was measured as standard length (SL) at 30 days old (mm) when fry were assigned to one of the two temperature treatments. Prior to analysis we used a Shapiro–Wilk test to determine whether early growth followed a normal distribution (function “shapiro.test”). Although the Shapiro–Wilk suggested the data were approximately normal, a *q*–*q* plot suggested evidence of right skew in the early growth data. Therefore, we used a log-transformation to minimize the skew. We determined the significance of the main effect and covariate with the function “Anova” in the package “car” (v. 3.1-3; Fox & Weisberg, 2019). We tested the significance of the dam effect using the function “ranova” in the package “lmerTest” (v3.1-3) (Kuznetsova, Brockhoff & Christensen, 2017). Comparisons to early growth rates measured in a previous study of *X. multilineatus* reared at 22 °C were made for size and age at sexual maturity (see below) but could not be made for growth rates per se, as the ages used for measuring early growth were not the same (14 days old–70 days old; Murphy, Goedert & Morris, 2014).

### Size and age at sexual maturity

We used nonlinear growth models to estimate the size and age at sexual maturity. However, we first determined which growth curve best fit the data by comparing growth rates based on the von Bertalanffy growth function (VBGF), Gompertz (Gomp) and a Logistic (Log) curves with the R package “fishmethods” and the function “growth” (Nelson, 2023). We estimated the growth curves in separate analysis for the two treatment groups. Growth curves based on the different functions were compared using a pairwise permutation test with the R function “growthlrt” in the package “fishmethods”. The growth function with the lowest residual sum-of-squares was considered the best fit. The logistic growth function had the lowest residual sum-of-squares and was the best fit compared to the von Bertalanffy and Gompertz functions for both cold and warm treatments (Table 1). Moreover, the logistic growth model best describes the pattern of growth displayed in *X. multilineatus*. Whereas the Gomp and VBGF models assume indeterminate growth, the Logistic curve assumes no additional growth once sexual maturity is attained. Next, we calculated a growth curve for each individual using the logistic growth equation. Values for standard length at days 30, 100, 210, and 330 days were used to estimate the growth curves. We used the asymptote of the curve produced for each individual from each treatment to estimate both size and age at sexual maturity using the function “SummarizeGrowth” in the package “growthcurver” (Sprouffske, 2020), because males in this species stop growing after sexual maturity (Kallman, 1989; Tompkins, 2024). The growth coefficients for each individual were used to assess the influence of temperature on individual growth rate.

**Table 1:** Comparisons of growth curves based von Bertalanffy (VBF), Gomperz (Gomp) and logistic growth functions (Log) for both cold (A) and warm (B) treatments. In the case of both treatments, the logistic growth function had the lowest residual sum-of-squares (RSS). *k* = growth rate.

**(A) Model for cold**	**RSS**	***k*± SE**
VBF: *L*inf*(1 − exp(−K*(*t* − *t*0)))	720.7	0.41 ± 0.04
Gomp: *L*inf*exp(−exp(−K*(age −*t*0)))	708.6	0.52 ± 0.04
Log: *L*inf/(1 + exp(−K*(age −*t*0))	702.5	0.39 ± 0.04
**(B) Model for warm**	**RSS**	***k*± SE**
VBF: *L*inf*(1 − exp(−K*(*t* − *t*0)))	858.0	0.21 ± 0.02
Gomp: *L*inf*exp(−exp(−K*(age −*t*0)))	828.6	0.30 ± 0.02
Log: *L*inf/(1 + exp(−K*(age −*t*0))	815.2	0.39 ± 0.02

We compared our estimates of size and age at sexual maturity to the measures from a previous study for males reared at an intermediate temperature (22 °C), including only the subset of males that had experienced a similar social context and came from a courter lineage (Murphy, Goedert & Morris, 2014). Diets in the previous study were both higher quality (Tetra-min Tropical Flakes 2×/day and bloodworms, 3×/week), and lower quality (Nishikoi Wheat Germ Koi Food, 2×/day) than the current study, age at sexual maturity was based on the development of the gonopodium, and size at sexual maturity was based on size when males stopped growing (Murphy, Goedert & Morris, 2014).

To determine the factors that could explain variation in size and age at sexual maturity, a linear mixed model (function “lmer” in the package “lme4”) was used. For size at sexual maturity, dam was included as a random effect, treatment as a fixed effect and both initial size and growth rate as covariates. For age at sexual maturity, dam was included as a random effect, treatment as the fixed effect, and initial size as a covariate. The models were run with the REML method to correct for degrees of freedom. The significance of each factor was determined using the function “Anova” in the package “car” (Fox & Weisberg, 2019). Determination of the significance of the random effect used the function “ranova” in the lmerTest package. We obtained *R*^2^ values using the “r.squaredGLMM” function in the “MuMIN” package.

### Mortality

We calculated the rate of mortality as the proportion of fry out of the total litter that did not survive to age 330 d. Individuals and dams that did not have siblings in both temperature treatments were removed from the analyses (*n* = 11 individuals from nine dams). A rate of mortality was calculated for each dam as the ((Initial number of fry − Final number of fry)/Initial number of fry) in each temperature treatment. We assessed the influence of treatment and growth rate on mortality using a linear mixed model. The model included treatment as a fixed effect, early growth as a covariate and dam as a random effect. The significance of each factor was determined with the function “Anova” in the “car” package. The contribution of the random effect, dam, was determined using a likelihood ratio test (LRT).

### Lifespan

We also compared the average life span of males from the two treatments through the end of the experiment (765 days), allowing us to compare the average age span for all males from the two treatments (*n* = 54) with a *t*-test.

All aspects of this study were approved under the Animal Care Protocol 12-L-042 through Ohio University IACUC. Full data set is available in the Supplemental Materials.

## Results

### Early growth

**Figure 2:**
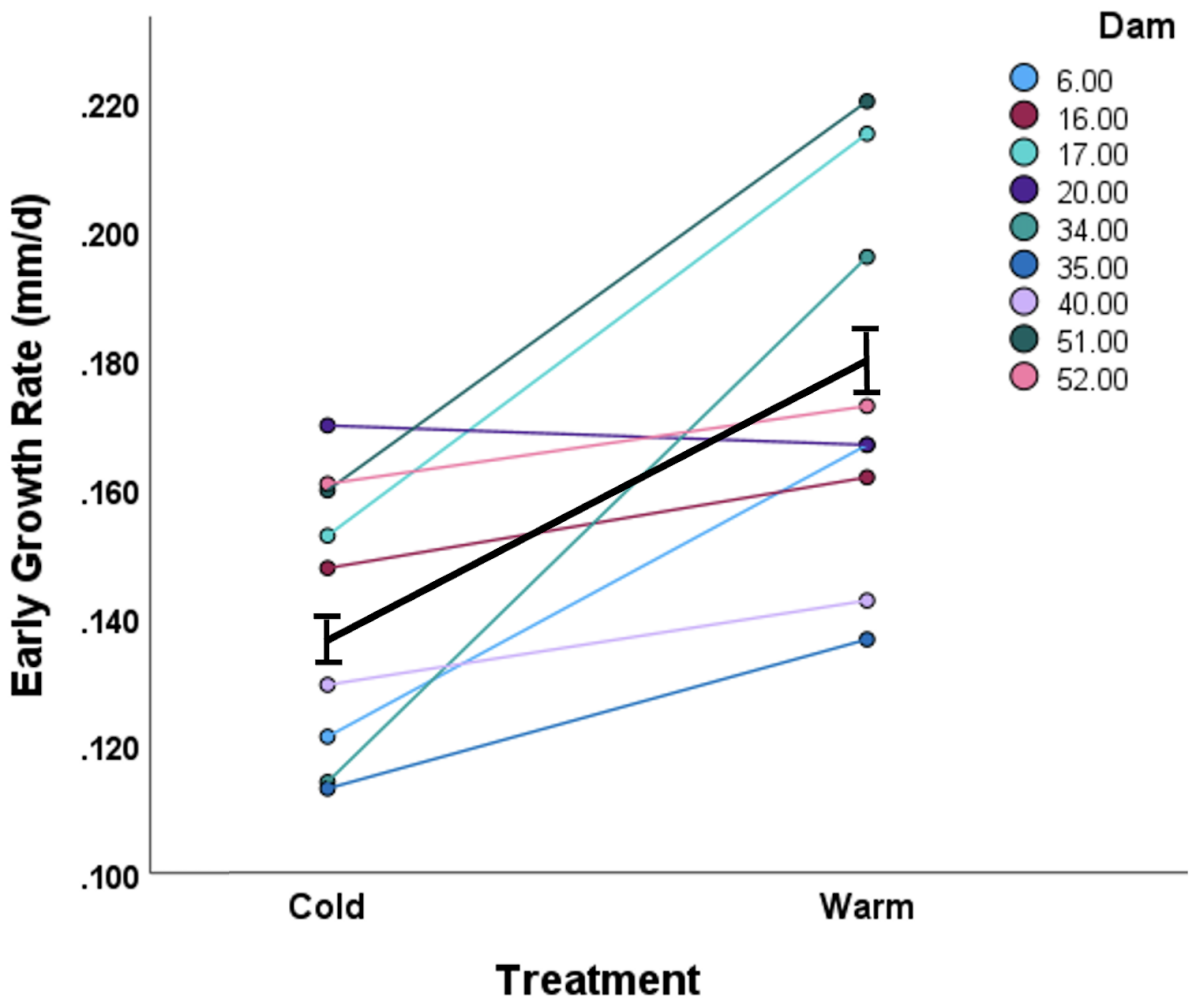
Reaction norms for early growth rates of *Xiphophorus multilineatus* males. The directional difference in early growth rate for offspring per dam between treatments is indicated; each family is indicated with a different colored line. Sample sizes per family/treatment can be found in the Supplemental Files. The mean difference between treatments is represented by black line with standard error bars.

Treatment was significant in explaining variation in early growth rate (*β* = 0.12; *χ*^2^ = 46.89, *P* < 0.001, *n* = 54) with faster early growth rates in the warm (mean = 0.176 ± 0.006 mm/d, *n* = 27) as compared to the cold (mean = 0.128 ± 0.003 mm/d, *n* = 27) environments. Initial size was not significant (*β* = −0.005, *χ*^2^ = 1.17, *P* = 0.28). Moreover, the dam effect was significant (LRT = 8.48; *p* = 0.0035) and explained 44.8% of variation in early growth rate as determined by the ICC. Variation in early growth rates by dam (averaged by full sibling) across treatment are portrayed in Fig. 2.

### Size and age at sexual maturity

**Figure 3:**
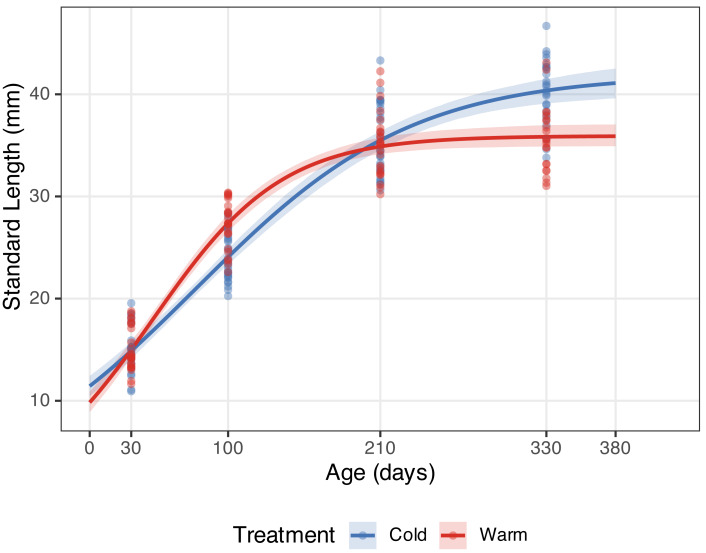
Logistic growth curves of *Xiphophorus multilineatus* courter males reared in the warm (red) andcold (blue) treatments. Shaded areas are the 95% confidence intervals generated using bootstrap estimates. Sexual maturity (*i.e.*, asymptote of curve) was at younger ages for males in the warm as compared to the cold, and the size at sexual maturity was smaller for warm as compared to cold.

Comparing the size and age of males that survived to sexual maturity across the two treatments, males from the warm treatment (*n* = 22) matured earlier (mean = 139 ± 8.1 d), but at a smaller size (mean = 35.7 ± 3.22 mm) as compared to males from the cold treatment (*n* = 24) that matured later (mean = 317 ± 8.1 d) and at a larger size (mean = 40.3 ± 3.22 mm) (Fig. 3). The factors that best explained variation in size at sexual maturity (*i.e.,* asymptotic male size, which corresponds with cessation of growth) included treatment (*χ*^2^ = 65.19, *df* = 1, *p* < 0.0001, *R*^2^ = 0.72), with individuals from the warm treatment attaining a smaller asymptotic size (Fig. 3). Initial size and dam are collinear, and when both are included, initial size did not contribute to explaining variation in asymptotic size (*χ*^2^ = 1.32, *df* = 1, *p* = 0.25). However early growth (*χ*^2^ = 13.03, *df* = 1, *p* < 0.001) and dam (*χ*^2^ = 7.01, *df* = 1, *p* = 0.008) did explained variation in size at sexual maturity. The factors that best explained variation in age at sexual maturity included treatment (*χ*^2^ = 91.93, *df* = 1, *p* < 0.0001, *R*^2^ = 0.72), but not initial size (*χ*^2^ = 3.17, *df* = 1, *p* = 0.08) or dam (Likelihood Ratio Test comparing model with and without random effect = 1.005, *p* = 0.32).

The average size for adult males from a previous study reared at 22 °C on the high quality diet (mean = 35.2 ± 0.74 mm, *n* = 9) and low quality diet (mean = 34.3 ± 0.43 mm, *n* = 19; Murphy, Goedert & Morris, 2014), were more similar to the adult size for males reared at the warmer temperature, but smaller than the average for males reared at the cooler temperatures in the current study. The average age for sexual maturity for males from a previous study of *X. multilineatus* reared at 22 °C on the high quality diet (mean = 172 ± 7.88 d, *n* = 7) and low quality diet (mean = 188 ± 7.06 d, *n* = 19; Murphy, Goedert & Morris, 2014), were in between the average age at sexual maturity for the males reared at the warmer and cooler temperatures in the current study. These results suggest that including an intermediate temperature treatment is unlikely to have modified the conclusions for our results in relation to size and age at sexual maturity comparisons.

### Mortality

Although the ICC revealed 50.3% of variation in mortality was attributable to dam (maternal effects), the linear mixed model analysis revealed insignificant variation in mortality attributable to dam effects (likelihood ratio test, *χ*^2^ = 2.21, *p* = 0.13). The disparity is likely due to the low number of dams included in this analysis (dams *n* = 9; offspring *n* = 1–4/per treatment), which limits the power to detect dam-level variance in mortality. Hence, the results for the influence of dam on mortality should be interpreted with caution. The effect of treatment was significant in explaining mortality (*χ*^2^ = 4.52, *p* = 0.033), but early growth rate was not (*χ*^2^ = 0.31, *P* = 0.58). Mean brood mortality in the warm treatment exceeded that in the cold treatment (warm = 0.62 ± 0.13, cold = 0.30 ± 0.12). By using the predicted age at maturity for individuals in each treatment calculated from the logistic growth function eight of the males across the total sample size (54) died before reaching sexual maturity (3, 11.1%, from the cold treatment and 5, 18.5%, from the warm).

### Lifespan

By the end of the experiment, the mean lifespan of males from the cold treatment (517 ± 39.4 d, *n* = 27) exceeded that of males from the warm treatment (408 ± 37.4 d, *n* = 27) (*t*-test = 2.0, *df* = 52, *p* = 0.052).

## Discussion

The effect of temperature on early growth rates, as well as age and size at sexual maturity of *Xiphophorus multilineatus* courter males, appears to follow the temperature-size rule. Males reared at warm temperatures grew faster as juveniles and matured earlier at smaller sizes than males from the cold treatment. Smaller male swordtails have lower mating success (Morris, Barta & Ryan, 1992; Rios-Cardenas, Tudor & Morris, 2007), as well as increased vulnerability to predation as adults (Basolo & Wagner Jr, 2004). However, given that the smaller sizes in the warm treatment were associated with faster growth rates which led to decreases in the age at sexual maturity, this would increase the probability of reaching sexual maturity given predation risk. Therefore, the results from this study predict a shift to faster growing, smaller courter males in this species due to global warming, while leading to a least one positive reproductive benefit (reaching sexual maturity sooner) in the face of several potential costs (Fig. 1). Below we discuss predictions for the responses of this species to global warming, as well as potential mechanisms involved in the responses we detected. We also consider how global warming could change the frequency at which the ARTs are evolutionarily stable, which depends on on whether the males from the sneaker alternative reproductive tactic (ART) also follow the temperature-size rule.

The split brood design of this study allowed for comparisons between treatments while controlling for differences in maternal provisioning as well potential genetic variation. There is extensive variation in adult male size within the courter males (see Lampert et al., 2010) known to be influenced by variation in copy number of the mc4r gene (Liu et al., 2020). Males from the same dam were likely to be equally provisioned prior to birth, and to the extent that they were full or half siblings (Luo et al., 2005) genetically similar through both dam and sire. Therefore, in addition to controlling for these influences between treatments, both aspects of maternal effects will need to be considered in those cases where dam was found to significantly influence the traits analyzed. Maternal effects due to resource provisioning are known to explain variation in early growth rates in live-bearing fishes (Reznick, Callahan & Llauredo, 1996; Lindholm, Hunt & Brooks, 2006; Kruuk et al., 2015). Female *X. multilineatus* fed a high-quality diet produced offspring that were initially bigger, grew faster and matured at a larger size than females fed a low-quality diet, suggesting increased resource provisioning by females with a higher quality diet (Murphy, Goedert & Morris, 2014). The daughters of *X. multilineatus* females that mated with courter males reached sexual maturity sooner than the daughters of females that mated with sneaker males, suggesting a maternal effect due to either provisioning (Rios-Cardenas, Brewer & Morris, 2013), and/or genetic influences other than Y-linked genes. However, the influence of maternal provisioning on body size was shown to be reduced throughout ontogeny in the live-bearing fish *Poecilia parae* and was attributed to a sharp rise in sire genetic variance for male body size (Lindholm, Hunt & Brooks, 2006). A reduced influence of maternal provisioning later in life suggests the potential for genetic influences to be responsible for the significant maternal effects on size at sexual maturity that we detected. The extent to which we detected dam related variation is due to maternal provisioning or genetic influences will require further study and will be important for predicting the extent to which adaptive responses to global warming will be short term *versus* evolutionary.

Variation in copy number of the melanocortin 4 receptor (*mc4r*) B alleles has been found to be associated with adult male size in *X. multilineatus* (Lampert et al., 2010; Liu et al., 2020) and yet the mechanism that triggers the onset of puberty is not known. The nonfunctional Y-linked *mc4r* copies in larger males act as dominant-negative mutations and are therefore thought to delay the onset of puberty, leading to variation in male size as males stop growing at sexual maturity. If we assume that at least some of the variation across males with different dams in this study is influenced by genetic variation of the sires (in the cases of full siblings), our results suggest a previous identified mechanistic hypothesis to test. We found that for the models that examined variation in size and age at sexual maturity, temperature treatment influenced both, but dam was only significant for body size. Therefore, it would be interesting to consider mechanisms in which the role for the *mc4r* gene in triggering sexual maturity is involved more directly with size as compared to age. One such hypothesis suggests that the triggering mechanisms for sexual maturity in fish involves the relative gill surface area (GSA for weight), which declines with size and influences relative O2 supply (Pauly, 2022). Given that *mc4r* has been found to be associated with appetite control in salmon (Kalananthan et al., 2020), increased weight could play a role in triggering sexual maturity. Future studies examining how variation in *mc4r* may be influencing this relative gill surface area threshold could provide a better understanding of how this gene influences the onset of puberty in *Xiphophorus* fishes.

In addition to higher mortality, fish reared in the warm had faster growth rates. Previous work with lab reared *X. multilineatus* found that courter males that had faster early growth rates had shorter adult lifespans (Murphy, Goedert & Morris, 2014; Weinstein et al., 2019). This tradeoff between faster growth and longevity has been found across taxa (Mangel & Stamps, 2001; Metcalfe & Monaghan, 2003). Even though the relationship across groups in the current study suggests a tradeoff between early growth rate and adult mortality, when controlling for treatment and dam in our models, our measure of early growth rate was not significant in explaining variation in mortality. While the power of our analysis was limited due to the number of dam included in the mortality analysis, there are two additional considerations that should be examined in future studies. First, it may be important to consider growth rates measured earlier than those we used in the current study to detect the tradeoff with mortality. Both the studies that detected this tradeoff in *X. multilineatus* used earlier measures (14–70 days Murphy, Goedert & Morris, 2014, 0–30 days Weinstein et al., 2019) as compared to the current study (30–70 days). Earlier growth rates are known to have larger impact on developmental errors, and the lack of an influence of the later growth rates we measured on mortality lend support to this finding. Second, in future studies it will be important to consider a potential confounding influence of dam, as the current study is the only study that examined variation in mortality across dams. In other words, it would be interesting to ask if increases in maternal provisioning could potentially counteract faster early growth rates, reducing the growth-mortality tradeoff.

The temperature-size rule broadly explains body size reductions observed among ectotherms correlated with recent warming trends (Daufresne, Lengfellner & Sommer, 2009; Forster, Hirst & Atkinson, 2012; Tian & Benton, 2020). However, while the temperature-size rule is ubiquitous, there are exceptions among ectotherms, particularly among insects and reptiles (Atkinson, 1994; Fischer & Fielder, 2002; Van der Have & De Jong, 1996; Sears & Angilletta Jr, 2004). There is evidence of divergence from the temperature-size rule over a relatively short period in the invasive Cabbage White Butterfly (*Pieris rapae*) in response to a warmer climate (Kingsolver et al., 2007). In this case, a southern population (North Carolina) responded to a warmer environment by attaining larger adult size with longer development time than cohorts reared at colder temperatures. The response detected across twelve species of salmonids to warmer temperatures found that more than two-thirds of populations examined increased in length over time (Solokas et al., 2023), and in a study where temperatures were experimentally modified in an enclosed coastal bay in the Baltic Sea, small bodied perch grew faster but increased in body size over 24 years (Huss et al., 2019). Not only do these examples challenge the hypothesis that declining body size is a universal response to warming, they suggest the potential for both short- and long-term adaptive responses as we have predicted based on the variation in reaction norms across dams for the courter males in *X. multilineatus* (Fig. 3).

Finally, future studies will want to consider how warming will affect the evolutionary equilibrium that maintains the two male ARTs (courter and sneaker) in *X. multilineatus*. While this question has been addressed in relation to ARTs that are not maintained due to equal fitness (condition-dependent polyphenism, Knell & Parrett, 2024), it has not yet been considered in a system where ARTs are genetically influenced and have equal fitness (*i.e., X. multilineatus*, Rios-Cardenas, Bono & Morris, 2018). The questions remains whether or not the sneaker males will also have a response of growth rate to temperature and either follow the temperature-size rule or not. Given enough variation in reaction norms and the potential for negative directional selection on growth rates for sneaker males (Abbott, Rios-Cardenas & Morris, 2019), we predict that sneakers will adapt to a warming environment by resisting an increase in growth rate and a decrease in size in lieu of the temperature-size rule, a pattern that has been detected in some other fish species (*i.e*., Solokas et al., 2023; Audzijonyte et al., 2020). While this scenario is narrow in scope, leaving out potential selection by warming on other important tradeoffs (Audzijonyte & Richards, 2018), the results detected for courter males suggest warming could alter the bimodal nature of these two ARTs by drawing courter males phenotypically closer to sneaker males.

## Conclusions

Within males of the courter ART in the swordtail *X. multilineatus*, the temperature-size rule applies. Courter males from the warm environment, on average, grew faster and reached maturity sooner at a smaller adult size than courter males from the cold environment. However, there appears to be variability among *X. multilineatus* courter males in response to being reared in different thermal environments due to dam, both in relation to the temperature-size rule and the growth rate-mortality tradeoff. The extent to which this variability is due to maternal provisioning as compared to genetic influences will be important to determine for predictions about the potential for this species to evolve adaptive responses in the face of rising temperatures. The impact of environmental warming on males from the courter ART in *X. multilineatus* will initially be to increase growth rates and decrease adult male size. The costs involved with this response will include reduced adult longevity and reduced mating success, but the benefit of an increased probability of reaching sexual maturity. The extent to which this pattern is detected in the future will depend on overall selection on these changes, as well as their ability to respond with either phenotypic plasticity or selection on genetic variation.

##  Supplemental Information

10.7717/peerj.21555/supp-1 Supplemental Information 1 ARRIVE Checklist

10.7717/peerj.21555/supp-2 Data S1 Data for analyzing variation in early growth rates, age at sexual maturity, size at sexual maturity and comparing longevity across treatments Dam = mother ID Treatment = Cold = 20 °C; Warm = 25 °C Fish ID = male ID Initial SL = standard length at 30 days old (when transferred to treatment) Early Growth = (SL 100 days −SL 30 days)/70 days Final Age = last age sampled; estimate of longevity Final SL = standard length measured from last photograph K = growth rate from curves Size at Maturity = based on asymptote of growth curves Age at Maturity = based on asymptote of growth curves.

10.7717/peerj.21555/supp-3 Data S2 Data for analyzing variation in mortality by families (dam) Dam = mother ID Treatment = Cold = 20 °C; Warm = 25 °C Early Growth (SL 100 days −SL 30 days)/70 days averaged across siblings Pooled Mortality = total number of siblings that died in each treatment prior to 330 days of age, divided by the total number of siblings reared in that treatment. *N* = number of siblings for this dam and treatment.

10.7717/peerj.21555/supp-4 Supplemental Information 4 Legend for Data Set

## Additional Information and Declarations

### Data Availability

The following information was supplied regarding data availability: The data is available in the Supplemental Files.
