# Supplementary material for: Predicting responses to warming temperatures for early growth rate, size and age at sexual maturity and mortality in male Xiphophorus multilineatus
Source: PeerJ. 2026 Jul 22;14:e21555. doi: 10.7717/peerj.21555 (PMC13401360; doi:10.7717/peerj.21555)
Supplement: Supplemental Information 4 [file peerj-14-21555-s004.docx]

Supplemental Materials

Data Files

Table S1. Data for analyzing variation in early growth rates, age at sexual maturity, size at sexual maturity and comparing longevity across treatments.

***Column Headings***

Dam = mother ID

Treatment = Cold = 20°C; Warm = 25°C

Fish ID = male ID

Initial SL = standard length at 30 days old (when transferred to treatment)

Early Growth = (SL 100 days – SL 30 days)/70 days

Final Age = last age sampled; estimate of longevity

Final SL = standard length measured from last photograph

K = growth rate from curves

Size at Maturity = based on asymptote of growth curves

Age at Maturity = based on asymptote of growth curves

Table S2. Data for analyzing variation in mortality by families (dam)

***Column Headings***

Dam = mother ID

Treatment = Cold = 20°C; Warm = 25°C

Early Growth (SL 100 days – SL 30 days)/70 days averaged across siblings

Pooled Mortality = total number of siblings that died in each treatment prior to 330 days of age, divided by the total number of siblings reared in that treatment.

N = number of siblings for this dam and treatment
